# The role of miR-145, miR-200c, and miR-21 in distinguishing between hyperplastic and adenomatous colon polyps and colorectal cancer prediction

**DOI:** 10.3325/cmj.2025.66.345

**Published:** 2025-10

**Authors:** Esra Guzel Tanoglu, Alpaslan Tanoglu, Samet Ebinc, Ismail Yılmaz, Muhammed Fevzi Esen

**Affiliations:** 1 Department of Molecular Biology and Genetics, Institution of Hamidiye Medical Sciences, University of Health Sciences Turkey, Istanbul, Turkey; 2 Experimental Medicine Research and Application Center University of Health Sciences Turkey, Istanbul, Turkey; 3 Division of Gastroenterology, Department of Internal Medicine, Bahçeşehir University, Istanbul, Turkey; 4 Department of Pathology, Sultan Abdulhamid Han Training and Research Hospital, University of Health Sciences Turkey, Istanbul, Turkey; 5 Department of Health Information Systems, Institution of Hamidiye Medical Sciences, University of Health Sciences Turkey, Istanbul, Turkey

## Abstract

**Aim:**

To assess whether miRNA expression can be a biomarker for distinguishing between hyperplastic and adenomatous polyps and colon cancer tissues.

**Methods:**

The study involved 40 colon adenocarcinoma (CA) tissue samples, 40 low-grade adenomatous (A) polyps, and 40 hyperplastic (HP) polyps. The samples were formalin-fixed and paraffin-embedded. Quantitative real-time polymerase chain reactions were used to determine the expression levels of miR-21, miR-200c, and miR-145.

**Results:**

miR-145 expression was significantly lower in the CA than in both the A and HP groups (*P* = 0.012 and *P* = 0.004, respectively) and in the A than in the HP group (*P* = 0.028). miR-200c expression was higher in the CA than in the A (*P* = 0.008) and HP groups (*P* = 0.009), while no difference was observed between the A and HP groups (*P* = 0.624). miR-21 showed a stepwise increase across the groups, with the highest levels in the CA, followed by the A and HP groups (*P* < 0.001 for both CA vs A and CA vs HP; *P* = 0.037 for A vs HP).

**Conclusion:**

The study revealed dysregulated expression of miR-145 and miR-21 in colon polyps and cancer tissue, showing that these miRNAs may be used to distinguish between hyperplastic and adenomatous polyps and predict colorectal cancer.

Colon cancer (CC) ranks third among all cancers in terms of incidence and second in terms of the number of deaths (1). Moreover, its prevalence and incidence have been continuously increasing worldwide (2,3), as have its mortality rates.

Adenomatous polyps are precancerous, while hyperplastic polyps are generally believed to be benign. Sessile serrated polyps are considered precancerous and cause 25%-30% of CC cases (4). The prevalence of colorectal adenomatous polyps varies considerably between countries and is associated with general CC occurrence rates in each country (5). Therefore, adenomatous polyps have to be detected early to prevent their progression to cancer (6). Early diagnosis of colorectal cancer (CRC) increases survival, reduces the likelihood of recurrence, and predicts treatment response (7). In this context, the histopathological examination of colon polyps is indispensable for an accurate diagnosis and follow-up in predicting progression to cancer. However, the invasive nature of colonoscopy has driven scientific research to explore non-invasive methods of the detection and evaluation of colon polyps (8).

MicroRNAs (miRNA) are non-coding RNA molecules approximately 18-25 nucleotides long. In cancerous cells, miRNAs supervise post-transcriptional gene expression as well as several cellular and developmental processes (9,10). They also regulate gene expression to control cellular proliferation and differentiation in cells. Depending on the properties of the mRNA in molecular pathways, miRNAs show either oncogenic or tumor-suppressive functions (11). Several miRNAs play an important role in tumorigenesis (12,13). Furthermore, they are crucial for cancer initiation, progression, and metastasis (14,15). There is insufficient literature data on whether miRNAs can be used to distinguish between hyperplastic and adenomatous polyps. Therefore, this study assessed whether miR-145, miR-200c, and miR-21 might be candidate biomarkers for distinguishing between hyperplastic and adenomatous polyps and CC.

## Materials and methods

### Tissue samples and laboratory data

The study was approved by the institutional review board of Sancaktepe Sehit Prof. Dr Ilhan Varank Education and Research Hospital. Formalin-fixed paraffin-embedded (FFPE) colon cancer tissue specimens and FFPE colon polyp samples obtained by polypectomy were acquired from the Department of Pathology of the University of Health Sciences, Sultan Abdulhamid Han Training and Research Hospital, Istanbul. All samples were obtained after receiving written informed consent from the patients. The study enrolled 60 women and 60 men aged 18-75 years. One polyp (≤10 mm diameter) from each participant was included. There were 40 adenocarcinoma (CA) tissue samples, 40 adenomatous (A) polyps (low grade), and 40 hyperplastic polyps (HP). Each group comprised tissue samples from 20 men and 20 women. Non-inclusion criteria were inflammatory bowel disease, familial adenomatous polyposis, hereditary nonpolyposis syndrome, other cancer types, and chronic inflammatory diseases and infections. Adenomatous polyps comprising tubular adenomas or HP and CC tissue specimens (adenocarcinoma) of pooled stages I-III (based on the degree of glandular differentiations) were included. Data on hemoglobin, platelets, white blood cell level, mean platelet volume, C-reactive protein (CRP), carcinoembryonic antigen (CEA), carbohydrate antigen 19-9 (CA 19-9), and neutrophil-to-lymphocyte ratio were retrospectively obtained.

### RNA extraction

The total RNA was isolated from paraffin colon cancer tissues using One Step-RNA Reagent (Bio Basic, Markham, Canada) according to the manufacturer’s protocol. The nucleic acid concentrations and purity values were measured with a spectrophotometer using BioSpec-nano (Shimadzu Biotech, Kyoto, Japan).

### cDNA synthesis and qRT-PCR

cDNA was synthetized using 30 ng RNA from each tissue specimen. Also, the miRNA-specific primer miRNA All-In-One cDNA Synthesis Kit (Applied Biological Materials, Richmond, Canada) was used according to the manufacturer’s recommendations. RNU43 was used for the normalization of miRNA expression analysis. Quantitative real time -polymerase chain reaction (qRT-PCR) was performed with Blastaq^TM^ 2X qPCR Master Mix (Applied Biological Materials) in a Bio-Rad CFX96 Real-Time System thermal cycler (Bio-Rad, Hercules, CA, USA), according to the manufacturer’s protocol. All samples were loaded into wells in duplicate. qRT-PCR was performed as follows: 1 cycle at 50 °C for 2-minute and at 95 °C for 10-minute, followed by 40 cycles at 95 °C for 15 seconds and 60 °C for 1 minute. The samples were amplified using the following primers: miR-145 forward 5′GTCCAGTTTTCCCAGGA′3, reverse 5′GAACATGTCTGCGTATCTC‘3; miR-21 forward 5′GCTTATCAGACTGATGTTG‘3, reverse 5′GAACATGTCTGCGTATCTC’3; and miR-200c forward 5′GTCTTACCCAGCAGTGT’3, reverse 5′GAACATGTCTGCGTATCTC’3. qRT-PCR results were evaluated according to the ΔΔCt method.

### *In silico* analysis

*In silico* analysis was performed with DIANA-miRPath v4.0 (16). In addition, the Kyoto Encyclopedia of Genes and Genomes (KEGG) was used to reveal the target interactions of miR-21, miR-200c, and miR-145. *P* values were determined for these interactions.

### Statistical analysis

Continuous variables are represented as mean ± standard deviation (SD). The Shapiro-Wilk-W test was employed to assess the normality of distribution. The normalized expression levels (ΔCt) for each miRNA were designated as follows: ΔCt1 for miR-200c, ΔCt2 for miR-21, and ΔCt3 for miR-145. The ΔCt value for each sample was calculated as ΔCt = Ct (miRNA) – Ct (RNU43), where a lower ΔCt value indicated higher miRNA expression. While miR-200c (ΔCt1) and miR-145 (ΔCt3) were normally distributed in all sample groups (*P* > 0.05), miR-21 (ΔCt2) was non-normally distributed (*P* < 0.05). One-way ANOVA was used to assess the differences between the groups in miR-200c and miR-145. Levene’s test was performed to assess the homogeneity of variances in groups, considering unequal variances across the groups (*P* < 0.05). For ΔCt2, the rank-based non-parametric Kruskal-Wallis one-way ANOVA test was used. Pearson correlation analysis was used to examine the associations between miRNA expression levels (ΔCt values) and laboratory test results within each group. A p value less than 0.05 was considered statistically significant. The analysis was performed with SPSS, version 20 (IBM Corp., Armonk, NY, USA).

## Results

**Figure 1:**
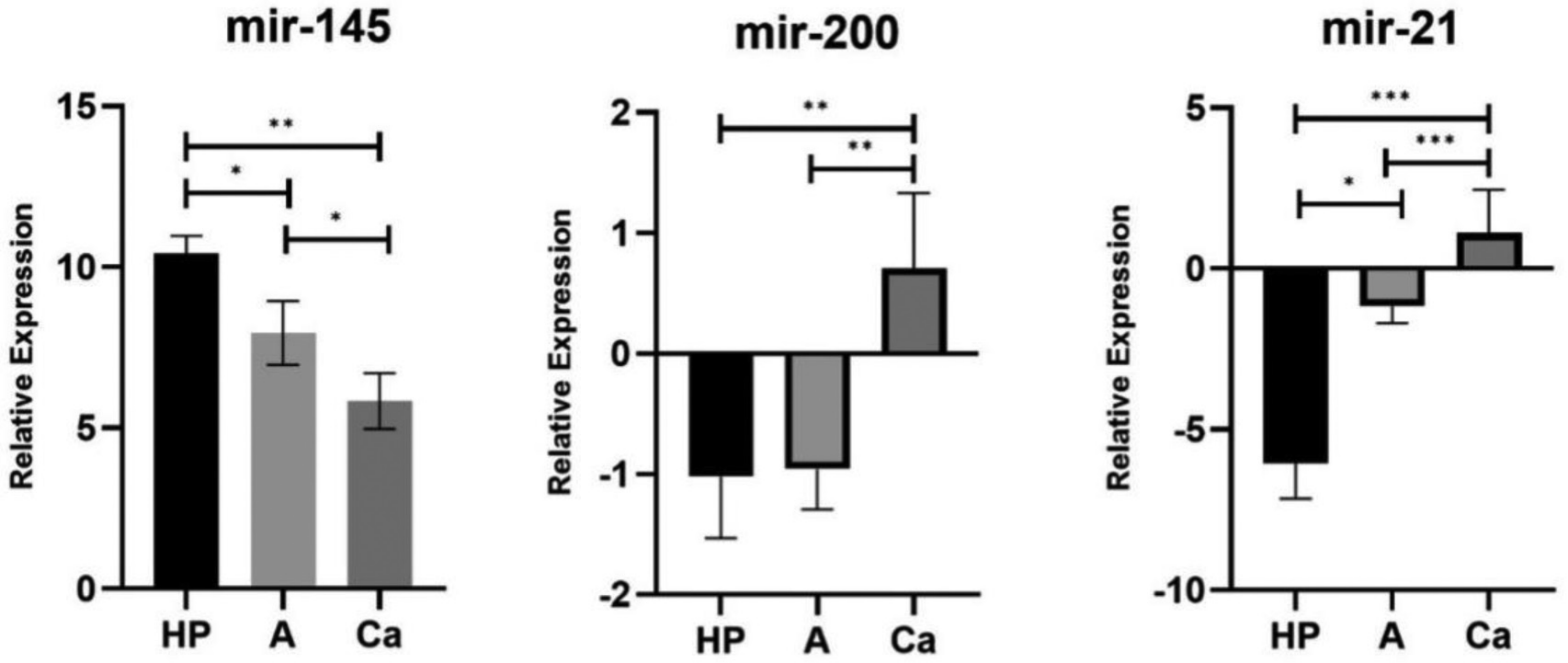
Micro-RNA levels in adenomatous polyps (A), hyperplastic polyps (HP), and colon adenocarcinoma (Ca) (*P < 0.05, **P < 0.01, ***P < 0.001).

The groups did not differ in terms of age and sex (*P* = 0.303 and *P* = 0.191, respectively). miR-145 expression level was significantly reduced in the CA group compared with the A (*P* = 0.012) and HP (*P* = 0.004) groups, and significantly reduced in the A compared with the HP group (*P* = 0.028). miR-200c was significantly higher in the CA than in both the A and HP groups (*P* = 0.008; *P* = 0.009), while no difference was observed between the A and HP groups (*P* = 0.624). miR-21 expression level was significantly higher in the CA than in the HP (*P* < 0.001) and A (*P* < 0.001) groups. It was also higher in the A than in the HP group (*P* = 0.037) (Figure 1). A moderate negative correlation was found between miR-21 expressions in the A and CA groups (r = -0.478; *P* = 0.033). There were no significant correlations between the pairs in any other group.

The mean CRP was significantly higher in the CA group compared with both the A and HP groups (*P* < 0.05), whereas there was no difference between the A and HP groups (*P* = 0.113). For platelets, the difference between the A and CA groups was significant (*P* < 0.001), but no difference was found between the A and HP (*P* = 0.123) and the CA and HP (*P* = 0.595) groups. Hemoglobin levels were significantly lower in the CA than in the A and HP groups (*P* < 0.01), but there was no difference between the A and HP groups (*P* = 0.102). Moreover, the groups did not significantly differ in terms of white blood cells level, mean platelet volume, and neutrophil-to-lymphocyte ratio (*P* > 0.05). Statistical analysis was not performed for CEA and CA 19-9 levels because there were not enough tumor marker data for the A and HP groups (Table 1).

**Table 1:** Descriptive statistics of laboratory test results for the adenomatous polyps (A) hyperplastic polyps (P), and colon adenocarcinoma (CA) groups

	A (n = 40)		CA (n = 40)		HP (n = 40)	
Laboratory parameter	mean	SD	mean	SD	mean	SD
C-reactive protein	10.02*	30.55	53.71**^†^**	68.05	7.50	26.25
White blood cell	9.09	6.39	9.37	5.50	7.84	3.73
Hemoglobin	12.95*	4.11	10.91**^†^**	2.97	13.46	2.72
Platelets	194.57*	142.62	280.88	159.38	248.93	78.87
Mean platelet volume	9.76	2.02	8.76	2.15	9.39	1.58
Carbohydrate antigen 19-9	NA	NA	44.73	10.81	NA	NA
Carcinoembryonic antigen	NA	NA	17.79	33.20	NA	NA
Neutrophil-to-lymphocyte ratio	9.50	22.83	11.60	35.80	9.07	14.29

*Statistically significant A vs CA.

**†**Statistically significant CA vs HP.

**Figure 2:**
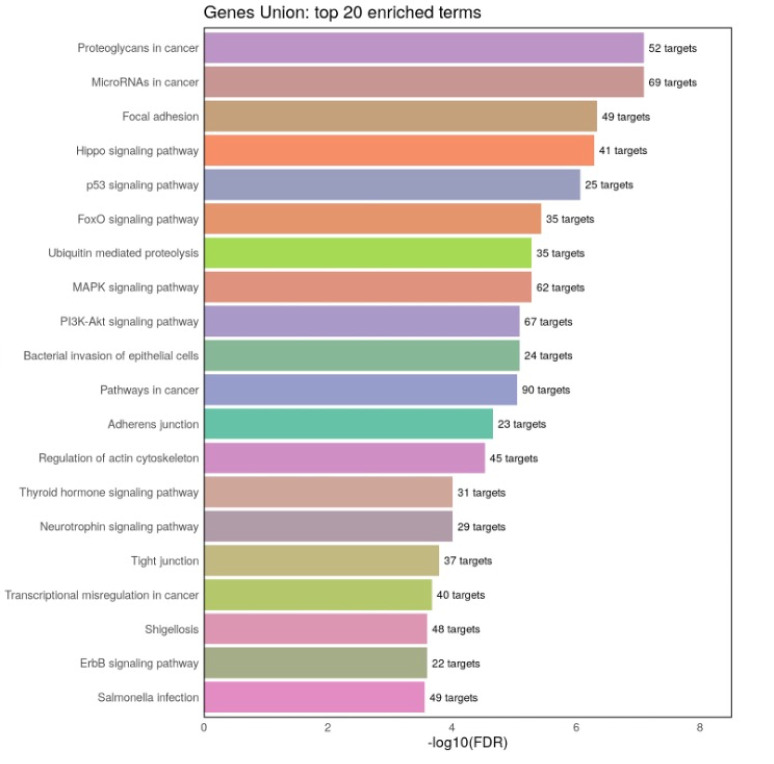
The significant clusters representation of miR-200c, miR-21, and miR-145 (FDR – false discovery rate).

MPV was positively correlated with miR-200c (r = 0.46; *P* = 0.04), miR-21 (r = 0.47; *P* = 0.04), and miR-145 (r = 0.46; *P* = 0.04) in the A group. Gene expressions did not positively correlate with any other laboratory parameter (*P* > 0.05). KEGG pathway analysis showed that miR-200c, miR-145, and miR-21 played a common role in the Hippo signaling pathway, p53 signaling pathway, proteoglycans in cancer, miRNAs in cancer, pathways in cancer, apoptosis, and colorectal cancer (Table 2). Figure 2 presents the significant targeted pathways clusters representation of miR-145, miR-200c, and miR-21.

**Table 2:** Overrepresented pathways that could be affected by miR-200c, miR-21, and miR-145

Kyoto Encyclopedia of Genes and Genomes pathway	*P* value	microRNAs
Proteoglycans in cancer	4.71^-10^	3
MicroRNAs in cancer	4.12^-10^	3
Hippo signaling pathway	5.93^-9^	3
p53 signaling pathway	1.25^-8^	3
Pathways in cancer	2.84^-7^	3
Apoptosis	0.0000335	3
Colorectal cancer	0.000196	3

## Discussion

This study showed that miR-145 and miR-21 may be used to distinguish between hyperplastic and adenomatous polyps and predict colorectal cancer. In other studies, miR-145 was found to be downregulated in breast, lung, stomach, ovary, and prostate cancer, playing an important role in cancer formation and progression (10,17-19). In CC, miR-145 expression was significantly decreased compared with the corresponding normal colon tissue samples (20). Additionally, in CC patients miR-145 levels were significantly reduced with lymph node metastases compared with those without lymph node metastases. Further, low miR-145 expression was associated with lymph node metastasis, distant metastasis, and TNM stage (20). One study reported that miR-145 and miR-619 showed high accuracy in distinguishing low-risk polyps without serrated histology from high-risk polyps (21). Conversely, the overexpression of miR-145 in colorectal cancer cell lines has been shown to inhibit cell proliferation, motility, and invasion (22). Another recent study showed considerably higher miR-145-5p expression in metastatic than in non-metastatic CC patients, and a correlation of elevated miR-145-5p levels and worse prognosis. As a result, miR-145-5p has been reported to act as a tumor suppressor in the initial phase of CC and to have oncogenic properties in the advanced stage of CC (23). In the current research, miR-145 expression levels were considerably decreased in CC and adenomatous polyps compared with hyperplastic polyps. Our results, consistent with the literature, suggest that miR-145 may be used as a biomarker in the distinction of hyperplastic and adenomatous polyps and in the follow-up of adenomatous polyps.

miR-200 regulates cell survival, proliferation, invasion, and metastasis in CRC (24). miR-200c expression increases in early-stage tumors of epithelial origin or chemotherapy-resistant subtypes, and decreases in breast, lung, and pancreatic tumors. In CRC, a high serum miR-200c level was positively associated with lymph-node and distant-organ metastasis and disease prognosis (25). On the other hand, miR-200c expression was significantly upregulated in CRCs compared with normal colon tissues (26,27) and was increased in high-grade and advanced-stage CRCs with lymphovascular invasion (27). In the current study, miR-200c was significantly upregulated in adenomatous polyps compared with hyperplastic polyps and in CRC tissues compared with all other polyps. These results indicate that miR-200c may be used as a biomarker in the diagnosis and follow-up of hyperplastic polyps, adenomatous polyps, and even early-stage CRC tissues.

miR-21 is oncogenic miRNA that affects the expression of many cancer-related genes such as *PTEN*, *RECK*, *TPM1*, and *PDCD* in colorectal, breast, lung, prostate, gastric, hepatocellular carcinoma, and glioblastoma cancers (28-30). It is highly expressed in CC specimens compared with adjacent normal tissues. High miR-21 levels are positively related to malignancy degree in CC patients and negatively linked with survival time (31). A high expression level of miR-21 in CC tissues may be a prognostic and predictive biomarker (32). Additionally, increased miR-21 expression in CC is related to vascular invasion, hepatic metastasis, and cancer stage, and is associated with poor prognosis. miR-21 also effectively predicts CC (33). Another study showed that circulation of miR-21 in CRC may be a potential diagnostic marker with good specificity and moderate sensitivity (34). In the current study, miR-21 levels were significantly increased in CC tissues compared with hyperplastic and adenomatous polyps. Thus, we believe that monitoring miR-21 levels in polyps may be meaningful in early detection of cancer.

To the best of our knowledge, this is the first study to simultaneously assess miRNAs as diagnostic biomarkers in hyperplastic polyps, adenomatous polyps, and CRC tissues. Although the expression levels of miR-145, miR-200c, and miR-21 significantly differed between polyp subtypes and colorectal cancer tissues, our study did not define the cut-off values that could be directly applied in clinical practice. Establishing such thresholds would require validation in larger, prospective cohorts using receiver operating characteristic curve analyses to determine their sensitivity, specificity, and predictive values. Nevertheless, our favorable results may contribute to the pool of knowledge on miRNAs as diagnostic biomarkers. In conclusion, miR-145 and miR-21 expression levels may be used as non-invasive biomarkers for distinguishing between hyperplastic and adenomatous polyps and predicting CRC.
